# Features of clinical, psychological and socio-demographic characteristics of day hospital patients in the context of psychiatric service reform

**DOI:** 10.1192/j.eurpsy.2021.1084

**Published:** 2021-08-13

**Authors:** V. Mitikhin, G. Tiumenkova

**Affiliations:** Department Of Mental Health Support Systems Research Centre, Mental Health Research Centre, Moscow, Russian Federation

**Keywords:** psychiatric, service, reform, psychosocial

## Abstract

**Introduction:**

The reform of the Moscow psychiatric service began in 2011 and was aimed at its optimization, reducing the inpatient level, actively introducing psychosocial rehabilitation, multidisciplinary teams of specialists and developing community-based forms of care. In 2016, the number of beds in day care hospitals in Moscow had doubled to rich 3500.

**Objectives:**

Analyze the dynamics of characteristics of day hospital patients and propose measures to improve the quality of medical rehabilitation care provided.

**Methods:**

Clinical and psychopathological, clinical and statistical, psychological, statistical of 337 schizophrenia patients discharged in 2010 and 2016.

**Results:**

A comparative analysis of the results obtained in 2010 and 2016 indicates a change in the clinical, socio-demographic and psychological characteristics of patients treated in the day hospital. In 2016, the proportion of early stage disease patients with endogenous mental disorders (F20-F29, according to ICD-10) increased; the age of patients and the proportion of patients with disabilities decreased; the proportion of patients with preserved working capacity increased, demonstrating low rates of compliance and motivation for treatment, but higher rates of neuro-cognitive functioning. In 2016, only a fifth of patients received complex psychosocial therapy.

**Conclusions:**

The modernization of the psychiatric service has improved the continuity between its inpatient and out-of-hospital units. To improve the quality of care in the day hospital and to prevent relapses of the disease, it is necessary to combine pharmacotherapy with complex psychosocial treatment followed by a long-term personalized management of patients with the patient’s families involvement.

